# Recruiting Small Manufacturing Worksites That Employ Multiethnic, Low-wage Workforces Into a Cancer Prevention Research Trial

**Published:** 2004-06-15

**Authors:** Elizabeth M. Barbeau, Lorraine Wallace, Ruth Lederman, Nancy Lightman, Anne Stoddard, Glorian Sorensen

**Affiliations:** Department of Society, Human Development and Health, Harvard School of Public Health, Boston, Mass. Center for Community-Based Research, Dana-Farber Cancer Institute; Center for Community-Based Research, Dana-Farber Cancer Institute, Boston, Mass; Center for Community-Based Research, Dana-Farber Cancer Institute, Boston, Mass; Center for Community-Based Research, Dana-Farber Cancer Institute, Boston, Mass; Center for Community-Based Research, Dana-Farber Cancer Institute, Department of Biostatistics, University of Massachusetts, Amherst, Mass; Center for Community-Based Research, Dana-Farber Cancer Institute, Department of Society, Human Development and Health, Harvard School of Public Health, Boston, Mass

## Abstract

**Introduction:**

Worksites, including those that employ multiethnic, low-wage workforces, represent a strategic venue for reaching populations at risk for developing cancer.

**Methods:**

We surveyed 197 small manufacturing worksites prior to an effort to recruit their workforces into a randomized clinical trial designed to test the effectiveness of a cancer prevention intervention among multiethnic, low-wage manufacturing workers. This paper assesses the external validity of the trial based on three factors: the percentage of potential trial sites excluded from consideration, the percentage of eligible worksites that adopted the trial, and worksite characteristics associated with adoption.

**Results:**

We found no statistically significant differences between worksites that adopted the trial and worksites that declined the trial with regard to employee demographics, anticipated changes in workforce size, and perceived importance and history of offering health promotion and occupational health and safety activities.

**Conclusion:**

Small manufacturing worksites present a viable venue for reaching multiethnic, low-wage populations with cancer prevention programs, although program adoption rates may be low in this sector. Worksites that adopted the trial are likely to represent worksites deemed eligible for the trial.

## Introduction

Cancer risk associated with health behaviors and carcinogenic occupational exposures is concentrated among working-class employees, individuals with less education, and some racial and ethnic groups (1-14). Worksites are a strategic venue for reaching these at-risk populations to reduce cancer risk. Cancer prevention research in small manufacturing worksites is particularly important because small manufacturing worksites employ roughly 42% of all manufacturing workers (15), are less likely to offer health promotion programs and protection from occupational health and safety hazards (16-26), and have been largely understudied (27). Furthermore, according to national survey data, some subgroups of the workforce, including nonprofessionals, blacks, and individuals with less education, were least likely to work for companies that offer health promotion programs to employees (28). When programs are available, blacks report the highest participation levels among all racial and ethnic groups (28). These data highlight the importance of conducting cancer prevention research in small worksites to address excess cancer risk among workers of lower socioeconomic position and racial and ethnic minorities.

Within studies such as this one, it is critical that researchers assess and report on worksite-level consent to participate, also known as adoption rate. Glasgow et al recently introduced the RE-AIM (Reach, Efficacy or Effectiveness, Adoption, Implementation, and Maintenance) model to assess intervention impacts (29). This model includes a measure for adoption, in which adoption is measured as the percentage of eligible worksites that adopt or test a health promotion program.

Adoption rates also are assessed for representativeness, or how well worksites that elected to participate in a program represent all eligible worksites. Representativeness is measured by comparing the characteristics of eligible worksites that adopt a health promotion program to eligible worksites that decline to adopt. Both assessments are critical to establishing the external validity of worksite-based studies, that is, the extent to which worksites recruited into trials represent other worksites (30). This type of rigorous assessment of external validity, however, is rare.

Bull et al recently evaluated the external validity of worksite health promotion studies (30). They reviewed intervention studies on dietary change, smoking cessation, and physical activity published in 11 leading journals during the five years from 1996 through 2000. They discovered that, among the 24 published studies, only six (25%) reported the percentage of eligible worksites that elected to participate in a program; only two (8%) reported exclusion criteria; and none reported on representativeness. In the two studies that reported exclusion criteria (30-32), the number of employees determined exclusion, and one also excluded worksites based on turnover rates and non-English-speaking employees (31).

Using the RE-AIM measures of adoption, our paper overcomes shortcomings of prior worksite health promotion studies to report on the process and results of worksite recruitment and worksite characteristics associated with program adoption in *Healthy Directions — Small Business* (HD-SB), a randomized, controlled cancer prevention trial among small-sized manufacturing companies employing multiethnic, low-wage workforces. The purpose of this paper is to assess the external validity of the trial, based on the percentage of potential trial sites excluded from consideration, the percentage of eligible worksites that adopted the trial, and the characteristics associated with adoption.

## Methods

### Overview

To assist the reader in interpreting the results of this report, we begin with an overview of the HD-SB cancer prevention trial itself and then focus on how we recruited worksites. The main question under investigation in HD-SB is whether or not a cancer prevention intervention that integrates health promotion and occupational health protection leads to significant mean improvements in workers’ consumption of fruits and vegetables, levels of physical activity, smoking cessation, and reductions in workers’ exposure to occupational carcinogens in small manufacturing worksites that employ multiethnic, low-wage workforces. Participating worksites are randomly assigned to either an intervention or a minimal intervention control condition. The intervention worksites receive an 18-month intervention focused on physical activity, diet, smoking cessation, and occupational health and safety. The control worksites receive only smoking cessation programs. Our institutional review board approved the trial protocol; employee participation in the trial is voluntary.

The intervention is an integrated health-promotion/health-protection model (33) based on social ecological theory (34,35). This model addresses both workers’ personal behaviors and the hazards of their work environments. Interventions are conducted at three levels: individual workers (e.g., health education about diet, physical activity, occupational health and safety), organization (e.g., worksite food options, programs to support worker physical activity such as lunchtime walking groups, occupational health and safety policies), and physical environment (e.g., reduction of carcinogenic exposures).

### Study population

The study population for this report is manufacturing worksites. We used the Dun and Bradstreet database to identify worksites with Standard Industrial Classification codes in the manufacturing group (Group D) that are located in and around a large northeast urban area in the United States and that employ between 50 and 150 workers. We selected manufacturing worksites because they are more likely than other worksites, such as those in the service sector, to use potential carcinogens in work processes. The worksite use of potential carcinogens allows us to intervene on cancer risks related to individual health behaviors as well as occupational exposures. We identified 224 companies in the Dun and Bradstreet database.

### Pre-recruitment survey measures

After identifying the 224 companies, we conducted a pre-recruitment worksite survey to determine eligibility for participation in the HD-SB trial. The pre-recruitment survey took place from March through August 1999. Our study eligibility requirements included the following:

- Employing a multi-cultural or multiethnic population (defined as 25% of workers being first- or second-generation immigrants or people of color).
- Having an employee turnover rate of less than 20% in the previous year.
- Being autonomous in decision-making power to participate in a study (if part of a larger parent company).

The survey asked respondents to indicate the total number of employees, the percentage of their workforce that was white and American-born, and the percentage of employee turnover within the last three years. To determine degree of autonomy, the survey asked respondents if they were able to make their own decision on program participation. In addition, the survey collected information about worksite characteristics (36) that we hypothesized would be positively associated with adoption, including perceived importance of and prior experiences with health promotion and protection programs and a positive financial outlook. The survey also asked respondents to rate their perception of the importance of health promotion and occupational health and safety activities on a 5-point Likert scale, to indicate if their worksite had previously offered such programs, and to say whether they anticipated increases, decreases, or no changes in workforce size in the next year (as an indicator of financial outlook).

### Data collection

Research staff placed phone calls to the 224 companies identified in the Dun and Bradstreet database to verify contact information. We then mailed the pre-recruitment survey to the CEO and director of personnel/human resources with a cover letter requesting their assistance in completing the survey as part of a research project to develop educational health promotion and health protection programs for manufacturing businesses. The letter contained no additional information about the research project. We contacted non-responders by telephone within two weeks, and research staff conducted the survey over the telephone. We attempted to reach non-responders at least 10 times by telephone. We attempted to reach both the CEO and director of personnel/human resources to maximize the potential for response. If both responded, we accepted the responses of the CEO only, thereby standardizing this aspect of data collection.

The mailed survey administration method yielded an unacceptably low response rate (11%; n = 24). As a result, we shortened the pre-recruitment survey and attempted to reach non-responders by telephone. The longer version of the survey asked about factors that would assist us in planning for intervention implementation, such as shift schedules, estimated percentage of employees who speak specified languages, and barriers and facilitators to worksite health promotion. We eliminated these questions to create the shortened survey (Appendix), which focused only on the measures, reported herein, that we hypothesized would relate to adoption. Research staff re-contacted non-responders and administered the shortened survey by telephone to either the worksite's CEO or director of personnel/human resources, increasing the response rate to 88%.

### Worksite recruitment

Once we deemed a worksite eligible to participate in the HD-SB trial based on the pre-recruitment survey, a member of the research staff contacted the survey respondent by telephone to describe the research trial and to assess interest in participating. If a company expressed interest, we conducted an in-person, on-site recruitment meeting to describe what would be required of participating companies, the specifics of the intervention condition, and the process of randomization to intervention or control condition. To participate, companies had to consent to allow employees to take baseline and final surveys, to allow research staff to conduct an industrial hygiene walk-through assessment of the worksite, and to conduct additional surveys with management on occupational health and organizational characteristics. If randomized to the intervention condition, worksites also were asked to

- Permit between five and 10 employees to meet monthly as part of an employee team designated to assist project staff with program implementation.
- Allow all employees at least 15 minutes per month during work time to attend project intervention activities.
- Have a HD-SB staff industrial hygienist consult with management to make plans for improving occupational health and safety conditions.

Once a company had agreed verbally to participate in the trial, a research staff member and company representative signed a letter of agreement stating participation requirements and indicating informed consent, or adoption. Recruitment took place from September 1999 through December 2000, with the first company beginning its 18-month intervention in September 2000 and the last company beginning its intervention in December 2000. All interventions were concluded by June 2002.

### Data analysis

Using data from the pre-recruitment survey, we determined the percentage of worksites that did not meet eligibility criteria and the percentage of worksites that met eligibility criteria and that adopted the program, and we compared the characteristics of companies that chose to participate in the trial with the companies that declined to participate. We calculated means and proportions to describe the sample and conducted Student *t*-tests (two-tailed) and chi-square tests for significance, with an alpha level of 5%.

## Results

Of the 224 worksites, 197 (88%) completed the pre-recruitment survey and 131 (66%) of these met the trial eligibility criteria. Among the 66 (34%) worksites deemed ineligible, reasons for ineligibility included not being engaged in manufacturing (n = 15), size of workforce (n = 23), lack of autonomy in decision making (n = 9), or insufficient percentage of workers being first- or second-generation immigrants or people of color (n = 19). Of the 131 worksites that met eligibility criteria, 26 consented to participate in the trial, for an adoption rate of 20%. The worksites recruited to the trial manufacture a range of products, including medical equipment, dog food, specialty pumps, textiles for the automobile industry, and electronics. Three of the worksites provide services to other businesses (laundry and printing).

Characteristics of eligible companies (n = 131) that adopted the intervention (n = 26) are compared with companies that declined (n = 105) (Table). On average, among all eligible companies, about half of all employees were persons of color and/or first- or second-generation immigrants to the United States; approximately one half of the worksites anticipated increasing the size of their workforce in the next year; approximately one quarter had a history of offering health promotion activities; approximately one quarter perceived such programs to be important (mean scores of 3.0 and 3.3 out of possible 5); most had a history of occupational health and safety activities; and most perceived these to be very important (mean score of 4.5 and 4.4 out of possible 5). Worksites that adopted the program were slightly more likely (differences not statistically significant) to have a larger percentage of white and American-born workers; to anticipate an increase in workforce size in the next year; to have offered health promotion and safety programs in the past year; and to perceive health promotion as important. We have no meaningful data on the small number of worksites that declined to complete the pre-recruitment survey (n = 27) and so cannot compare them to those that did.

An additional seven worksites consented to participate but withdrew prior to the start of the intervention (categorized as decliners in presented data), citing concern about lack of time to participate in the trial given increasingly tight production schedules. These seven companies were also slightly more likely to perceive health promotion as important and to have offered health promotion programs in the past, compared to other eligible worksites (differences not statistically significant). Later in the trial, one worksite withdrew from the intervention condition and another withdrew from the control condition; both cited lack of time as reason for withdrawal.

## Discussion

This paper reports on the process and outcome of our efforts to recruit small manufacturing worksites employing multiethnic, low-wage workforces into a cancer prevention intervention trial. Trial eligibility criteria excluded about 34% of worksites responding to our survey. Among eligible sites, 20% (26 of 131) adopted the program, a rate similar to other cancer prevention studies (13,33,37). An additional seven worksites initially consented but withdrew very early in the trial. Among worksites eligible to participate, we observed no statistically significant differences between those that consented and those that declined to participate in the trial with regard to workforce composition, anticipated expansion of the workforce (financial outlook), and perceived importance and history of heath promotion activities and occupational health and safety programs. In sum, we found that the racial and ethnic composition of the workforce, financial outlook, and perceived importance and experience with health programs were not barriers to adoption in cancer prevention trials in this sample of worksites.

The study had a few limitations. First, the survey relied on self-reports by a worksite representative, and we did not attempt to validate the information provided. Second, using the RE-AIM measures, we attempted to assess worksite participation in a cancer prevention research trial as a proxy measure for adoption of a cancer prevention program. Participation in a research trial is not the same as adoption of a program. And finally, our pre-recruitment survey did not contain measures that allowed us to characterize differences between adopters and decliners, suggesting that additional measures may be needed, the development of which might rely on qualitative, open-ended questions on factors that promote or inhibit adoption. The survey administrators noted anecdotally that employer reasons for adoption included having a family member with a history of cancer; viewing participation as a low-cost, value-added benefit for employees during a time of tight labor markets; wanting to take advantage of our occupational health and safety expert consultations; and believing that a healthy workforce is a more productive one. Common reasons noted by employers for declining to participate were lack of time and poor labor-management relations. These reasons may form the basis for distinguishing adopters and decliners in recruitment surveys for future trials.

Our findings have several important implications for the HD-SB trial and for other future worksite-based trials. First, although our adoption rate was 20%, a systematic assessment of the adoption rate using the RE-AIM framework indicates strong external validity for HD-SB trial findings: we found no significant differences between eligible worksites that adopted the cancer prevention trial and those that declined. We may generalize the findings of our main trial to other small manufacturing businesses that are located in urban areas and employ multiethnic, low-wage workers. The application of the RE-AIM measures for worksite adoption used here represents a key strength of our trial: few prior studies have reported explicitly on the percentage and representativeness of worksites that are willing to adopt or try a health promotion program (32). Second, the results provide guidance to future researchers and practitioners in estimating likely rates of adoption and early withdrawals. When recruiting small manufacturing worksites, which may be particularly vulnerable to volatile economic conditions and production schedules, it may be necessary to recruit additional worksites to allow for early withdrawals and avoid threatening the trial’s statistical power. A related point is that when attempting to reach worksites to assess eligibility for recruitment, researchers ought to use a short survey instrument that they can administer conveniently, preferably by telephone. Third, the high mean level of reported importance of occupational health and safety programs among all eligible worksites is noteworthy, suggesting that these programs may represent an attractive intervention component for small manufacturing businesses. This level of interest in health and safety has not been evident in studies of larger manufacturing worksites (33,37).

Recruitment for this trial took place within a larger social context: the decline of the U.S. manufacturing sector. U.S. manufacturing companies often are in precarious financial situations, or they may perceive that they have too little time to commit to a health promotion trial. On the other hand, they may view such an endeavor as a “free” resource. Our anecdotal data support both of these hypotheses, which can be subjected to rigorous assessment in future trials.

Reducing racial/ethnic and class-based health disparities is a major focus for the U.S. Public Health Service (12,38). Intervention research is essential to developing effective methods for reducing the disproportionate cancer risk associated with health behaviors and occupational exposures among immigrant, multiethnic and multi-racial, less-educated, and low-wage workers. Our results indicate that small manufacturing worksites are a viable community-based channel for reaching low-wage, multiethnic populations with cancer prevention programs, but that we can expect low adoption rates within this sector. Future intervention studies in these settings need to address the concerns of small businesses and to assess systematically the worksite characteristics that promote participation in trials and, ultimately, program adoption.

## Figures and Tables

**Table:** Comparison of Characteristics of 131 Eligible Worksites That Adopted or Declined Cancer Prevention Intervention for Employees, Northeastern United States, 2000^a^

**Worksite Characteristic**	**Declined Intervention** **n = 105**	**Adopted Intervention** **n = 26**
Mean percentage of workforce white and American-born	52.2%	60.6%
Proportion that anticipate increase in number of employees in next year	49.0%	53.9%
Proportion that offered health promotion programs in past year	24.8%	26.9%
Proportion that offered safety programs in past year	84.6%	88.5%
Mean perceived importance of worksite health promotion programs in company (1 = low; 5 = high)	3.0	3.3
Mean perceived importance of worksite safety programs in company (1 = low; 5 = high)	4.5	4.4

a No differences were found to be statistically significant, based on Student *t*-tests (two-tailed) and chi-square tests.

## Appendix

**Telephone Survey of Small Manufacturing Worksites That Employ Multiethnic, Low-wage Workforces, Northeastern United States, 1999**

Hello, my name is ______________________. I am calling from Dana-Farber Cancer Institute. We recently sent your company a questionnaire for a project we are conducting with small businesses in the Boston area. The questionnaire was called the “Health Survey of Small Businesses in Massachusetts.” We have reviewed the survey and have made changes to shorten it. Since we did not receive a completed survey from your company, would you be able to take about five minutes now to answer a few questions?

**Today’s Date:**

**Your Company’s Name:**

**Your Name:**

**Your Title:**

**Your Phone Number:**

**Your Fax Number:**

1. Do manufacturing or production operations go on at this worksite? (Yes/No)
2. About how many permanent employees working 20 hours or more per week are there in your company? Do not include temporary workers. (Total number)
3. About how many of those employees would you say are blue collar or directly involved in the manufacturing or production process? (Number)
4. About how many are piece workers? (Number)
5. Approximately what percentage of your workforce is represented by union(s)? (Percentage)
6. About what percentage or your workforce is white/American-born? (Percentage)
7. Do you anticipate your workforce will increase, downsize, or have no change in the next year? (Check one only)
8. In the past year, has your company offered any health promotion programs? (Yes/No) Check all that apply. Use the following list as prompts:
  - Nutrition classes
  - Exercise classes
  - Weight control classes
  - Health fairs
  - Smoking cessation classes
  - Safety Programs
  - Other (text)
9. In the past year, has your company offered any safety programs? (Yes/No)
10. About what percentage of your employees are currently covered by any amount of company paid health insurance? (Percentage)
  **If you are talking to the Human Resource Director, skip to Question #12.**
11. How important do you think it is to have worksite health promotion programs in your company? For example, nutrition, exercise classes, smoking cessation programs or material.
  Not at all important			Very Important	
  1	2	3	4	5
12. How important do you think it is to have worksite safety programs in your company?
  Not at all important			Very Important	
  1	2	3	4	5
13. In your opinion, how important does your **company management** think it is to have worksite health promotion programs in your company? For example, nutrition, exercise classes, smoking cessation programs or material.
  Not at all important			Very Important	
  1	2	3	4	5
14. In your opinion, how important does your **company management** think it is to have worksite safety programs in your company?
  Not at all important			Very Important	
  1	2	3	4	5
  I would like to thank you for your participation in the Health Survey of Small Businesses. One of the purposes of this survey is to identify potential participants for the Cancer Prevention in Small Businesses project, funded by the National Cancer Institute. The goal of the project is to develop a national model for worksite cancer prevention. The study offers two years of health programming provided by experienced staff at no cost to you. We will focus on healthy eating, increased physical activity, and safety issues.
15. Are you able to make the decision to participate in a program like this one on your own? (Yes/No) Who else would have to be consulted?

**Thank you for your participation in this survey.**
